# Differential Influence of Physical Activity on Cardiopulmonary Performance and Stroke Volume Assessed at Cardiopulmonary Exercise Test in Pectus Excavatum: A Pilot Study

**DOI:** 10.3389/fphys.2022.831504

**Published:** 2022-02-03

**Authors:** Lorenzo Casatori, Alessio Pellegrino, Antonio Messineo, Marco Ghionzoli, Flavio Facchini, Alessandra Modesti, Pietro Amedeo Modesti

**Affiliations:** ^1^Department of Clinical and Experimental Medicine, School of Medicine, University of Florence, Florence, Italy; ^2^Sports Medicine Unit, Careggi University Hospital, Florence, Italy; ^3^Department of Pediatric Surgery, Meyer Children’s University Hospital, University of Florence, Florence, Italy; ^4^Department of Biomedical, Experimental and Clinical Sciences “Mario Serio”, University of Florence, Florence, Italy

**Keywords:** exercise training, physical activity, cardiopulmonary exercise testing, O_2_ uptake, stroke volume, metabolic equivalent, pectus excavatum

## Abstract

**Background:**

Exercise training increases muscle VO_2_ by increasing O_2_ transport and O_2_ uptake while cardiac output increase might be limited by the conformation of the chest in subjects with pectus excavatum (PE).

**Aims:**

The aim of the present study was to investigate the influence of physical activity (PA) on functional parameters of cardiopulmonary performance and stroke volume obtained at Cardiopulmonary Exercise Test (CPET) in PE.

**Methods and Procedures:**

A cohort of adolescents (15 with PE and 15 age- and sex-matched healthy controls, HC) underwent Cardiopulmonary Exercise Test (CPET) and administration of the International Physical Activity Questionnaire – Short Form (IPAQ-SF) with estimation of weekly PA (METs h^–1^⋅week^–1^). Determinants of CPET parameters were investigated with multivariable linear regression analysis.

**Results:**

As expected, when compared to HC, PE had lower VO_2_ max (37.2 ± 6.6 vs. 45.4 ± 6.4 mL⋅kg^–1^⋅min^–1^, *p* < 0.05), and VO_2_/HR max (O_2_ pulse, 12.1 ± 2.4 vs. 16.2 ± 3.6 mL⋅min^–1^⋅bpm^–1^, *p* < 0.05). Importantly, physical activity level was a predictor of VO_2_ max (adjusted for sex, body mass index, FEV_1_%, and presence of PE, β = 0.085; 95% Cl 0.010 to 0.160, *p* = 0.029) whereas O_2_ pulse was independent from PA level (β = 0.035; 95% Cl −0.004 to 0.074).

**Conclusion:**

Physical activity is a determinant of VO_2_ max (cardiopulmonary performance), whereas it appears not to affect O_2_ pulse (a measure of stroke volume at peak exercise) related to constrained diastolic filling in PE.

## Introduction

Pectus excavatum (PE) is the most common congenital chest wall deformity of childhood. It occurs with an incidence of approximately 1 every 300–400 children (Neviere et al., 2011) and it occurs more frequently in boys (1:5) (Jaroszewski et al., 2010). Pectus excavatum begins to appear in childhood and manifests itself more frequently during puberty.

Although initially considered only an esthetic problem, it gradually emerged that this deformity can also cause functional disorders (Shneerson, 1998). Depending on the severity of the malformation, pectus excavatum can cause psychological, anatomical and physiological disorders of varying degrees (Alaca and Yüksel, 2021).

Outward appearance can become a major problem in adolescence, especially for those who show visible disfigurement (Steinmann et al., 2011). This can be reflected in choosing clothing or in avoiding social and sporting activities (Ji et al., 2011; Alaca and Yüksel, 2021). Limitation of activities can lead to physical deconditioning. Rowland et al. (2005) showed that adolescents with moderate to severe PE have lower endurance performance, expressed as the highest workload achieved, than healthy controls. In the most serious cases the dislocated sternum may also compress heart and lungs with a resultant stroke volume reduction, primarily caused by constrained diastolic filling. Both the limitation of physical activities and anatomical alterations finally result in real physiologic impairment with a reduction in exercise capacity and VO_2_ max, predominately because of impaired cardiovascular performance, rather than ventilatory limitation (Malek et al., 2003; Cavestri et al., 2010).

Deciding whether a patient can benefit from exercise-based treatment or should undergo surgery is crucial. Surgery repair of pectus excavatum is associated with postoperative pain (Archer et al., 2020; Uhl et al., 2020) and requires an important commitment on the part of the patient and his family. The minimally invasive Nuss surgical procedure is currently preferred for PE repair in children and adolescents with moderate and severe chest wall depressions (Morshuis et al., 1994; Haller and Loughlin, 2000; Sigalet et al., 2007; Neviere et al., 2011, 2013; Kelly et al., 2013; Maagaard et al., 2013; O’Keefe et al., 2013). Conservative treatments, based on the promotion of physical activity (PA), are preferred for patients with milder deformities (Abid et al., 2017).

Anatomical severity of PE is commonly measured by Haller Index, the ratio of the transverse diameter of the rib cage to the minimum distance of the lower sternum from the vertebra (Haller and Loughlin, 2000). However, the lack of predictive value of the index on the effects of surgery indicates that simple anatomical measurement is not sufficient to define the complex structural changes of the PE (Morshuis et al., 1994; Tang et al., 2012; Satur et al., 2021). With the cardiopulmonary test, which allows an integrated evaluation of the respiratory and circulatory parameters, an impairment of both functional capacity (VO_2_ max) and cardiac output (O_2_ pulse) was highlighted in patients with PE (Morshuis et al., 1994; Sigalet et al., 2007; Tang et al., 2012; Maagaard et al., 2013; O’Keefe et al., 2013). However, no correlation between the PA levels and cardiopulmonary impairment has been described in PE. The question has particular clinical significance both for indications for surgical intervention and for the promotion of PA in this group of patients. The promotion of PA could indeed enhance the functional capacity (VO_2_ max) but would probably have limited effects on cardiac output.

Aim of this pilot study is to explore the possible differential influence of physical activity on cardiopulmonary performance and stroke volume parameters obtained at Cardiopulmonary Exercise Test (CPET) in PE.

## Materials and Methods

### Patients Investigated

In total, 15 consecutive patients (4 females, 11 males) with PE, referred to our Unit for functional evaluation between March 2020 and August 2021 were investigated. The diagnoses were established by a thoracic surgeon (AMe) with 30 years’ experience. All the patients were in good health and had no evidence of cardiac or respiratory disease; none were taking medication that would affect cardiorespiratory fitness. Criteria for exclusion were severe lung disease, pre-existing heart disease, or an underlying causative syndrome such as Marfan’s disease, previous repair of pectus excavatum by any technique; previous thoracic surgery; congenital heart disease or pregnancy. The healthy control (HC) group consisted of 15 age- and sex-matched adolescents who responded to a local advertisement. The study was conducted according to the guidelines of the Declaration of Helsinki, and approved by the Tuscany Region clinical research ethics board (Comitato Etico Regionale per la Sperimentazione Clinica della Regione Toscana; protocol code 15840_bio, date of approval February 24, 2020). All participants provided written informed consent. All participants went through the same examinations and tests throughout the study.

### Evaluation of Physical Activity Levels

An evaluation was made using the International Physical Activity Questionnaire-Short Form (IPAQ-SF) (Hallal and Victora, 2004). The questionnaire calculates the metabolic equivalent (MET) value, and considers the frequency, duration (minutes), and intensity of physical activities performed for at least 10 min. over the last seven days. The questionnaire consists of four parts: (1) vigorous-intensity activity (requiring hard physical effort that makes breathing much more frequent than normal) such as lifting heavy objects, doing aerobics, or cycling at a certain speed, including how many minutes per day; (2) moderate-intensity activity (requiring moderate physical effort that makes breathing a little more frequent than normal), such as carrying light weights, riding a bike at a regular pace or playing tennis doubles, but not walking; (3) walking, divided in slow, moderate and fast pace; (4) time spent sitting, including reading, watching television, studying and video games. The total volume of PA accumulated over a week, calculated by multiplying the duration of the reported category by its assigned MET value, was expressed as METs h^–1^⋅week^–1^. The mean intensity of light PA, moderate PA, and vigorous PA was assigned as 3, 4, and 8 METs, respectively (Wahid et al., 2016; Cheng et al., 2018; Rosselli et al., 2020).

The IPAQ-SF is a valid (*r* = 0.67) and reliable tool (rho = 0.77–1.00) (Craig et al., 2003), that is recommended for assessing PA in young and middle-aged adults (15–69 years) [Mannocci et al. (2018); *The International Physical Activity Questionnaire, Available from: Guidelines for Data Processing and Analysis of the International Physical Activity* (IPAQ, 2021)].

### Cardiopulmonary Exercise

Each participant was asked to refrain from strenuous exertion the day before the test day and to refrain from eating solid foods or carbohydrate-rich drinks in the 3 h prior to the test. The test was performed in the morning in controlled environmental conditions (temperature 18–24°C, relative humidity 30–60%). Prior to exercise testing, body mass and body height were measured using an electronic scale. Body Mass Index (BMI, kg ⋅m^–2^) was calculated as the body mass divided by body height squared. Forced vital capacity (FVC), forced expiratory volume in 1 s (FEV_1_), and Tiffeneau index (FEV_1_/FVC) were also measured before the exercise test and expressed in percentage of the predicted value.

A symptom-limited CPET progressive ramp protocol was performed on an electronically braked cycle ergometer (Ergoline). Briefly, participants were equipped with an oronasal face mask connected to a metabolic cart (COSMED Quark CPET). CO_2_ exhaled and O_2_ consumed were measured using the breath-by-breath method. The workload ramp was individualized to achieve maximum exertion within 8 to 12 min. according to the recent recommendations reporting that VO_2_peak is maximal in 10-min ramp protocols (Paridon et al., 2006; Takken et al., 2017).

Selection of the workload ramp was based on predicted values for height (Godfrey et al., 1971). Minimum watt increase (1, 2, or 5 watt) for each ramp was set up to achieve the most linear increase possible and therefore the more uniform physiological responses. After 3 min of rest, patients performed a 3 min pedaling warm-up without load at 50 rpm. When the incremental workload started, patients were asked to pedal at 60–80 rpm until muscle exhaustion. The test was terminated when participants were no longer able to maintain pedaling frequency despite verbal encouragement. The test was considered maximal when at least 2 of the following conditions were reached: (1) peak respiratory exchange ratio (RER) ≥ 1.10; (2) HR max ≥ 85% of age-predicted maximum; (3) a plateauing of oxygen uptake (increase < 150 mL⋅min^–1^) in VO_2_ over the last 30 s of the test. Tests were interrupted if signs of cardiovascular distress were present (ventricular arrhythmias, falling blood pressure, dizziness, etc.). A twelve-lead ECG, and oxygen saturation were continuously monitored.

Oxygen Consumption (VO_2_), Carbon dioxide Production (VCO_2_), Tidal Volume (VT), Breathing Frequency (BF), Ventilation (VE), Heart Rate (HR) and Work Rate (WR) were measured throughout the test. The ventilatory Anaerobic Threshold (AT) was determined using the V-slope and the ventilatory equivalent approaches. Other variables were the ratio of VO_2_ and heart rate (VO_2_/HR, termed O_2_ pulse, a measure of stroke volume), oxygen-uptake to work-rate relationship (VO_2_/W slope, a measure of work efficiency), the product of VO_2_ max (in milliliters per kilogram per minute) by systolic blood pressure (VO_2_ max x SBP max, a measure of peak circulatory power).

Z score values were calculated by adopting the prediction model developed by Blanchard et al. (2018) assessing the relationship of the dependent variable (VO_2_ max) with three independent variables, height, body mass, and age/sex.

### Statistical Considerations

Statistical analyses were performed using SPSS 27 software (IBM, Armonk, NY). Sample size determination and power calculations were made for the volume of oxygen uptake/kg (VO_2_). With an estimated standard deviation of 20%, a difference between the 2 groups of 20%, an alpha of 0.05 and a power of 0.80, the number necessary for investigation was 15 subjects for each group.

All continuous variables are expressed as mean ± SD, unless specified. A paired, two-tailed Student’s *t*-test was used for comparison for continuous variables. Multivariable linear regression analysis was used to analyze the correlations between variables. Independent variables were sex, BMI, FEV_1_%, and presence of pectus excavatum; VO_2_ max, VO_2_/HR max or VO_2_/W slope as dependent variable. Assuming that the dependent variable is predicted by the set of 5 predictors and that the squared population multiple correlation coefficient is 0.30, that is that the 5 predictors account for 30% of the variance of the dependent variable, the sample size is *n* = 30 subjects (power 0.66).

A difference of *p* < 0.05 was considered statistically significant.

## Results

There were no significant inter-group differences in age or gender. Body mass index was significantly lower in the PE group compared to the HC group. No difference was observed between patients and controls in the level of habitual activity; more precisely, in the PE group, time spent per week in vigorous or moderate PA, as well as the total METs h^–1^⋅week^–1^ were not significantly different from those in the HC group (Table 1).

**TABLE 1:** Demographic characteristics and biometric data of study participants.

	Pectus excavatum	Healthy controls	p=
Boys/Girls (n/n)	11/4	11/4	_
Age (years)	15.5 ± 1.6	15.5 ± 1.5	0.907
Height (cm)	175.5 ± 9.7	173.1 ± 9.1	0.480
Weight (kg)	58.5 ± 7.2	65.1 ± 10.8	0.062
Body mass index (kg ⋅ m^–2^)	19.0 ± 2.1	21.6 ± 2.2	0.002
HR (bpm)	99.1 ± 23.3	83.5 ± 15.9	0.040
SBP (mm Hg)	110.3 ± 11.1	115.3 ± 13.0	0.267
DBP (mm Hg)	73.3 ± 9.8	70.7 ± 8.0	0.420
Vigorous activity (min ⋅ week^–1^)	79.3 ± 87.7	136.0 ± 125.0	0.172
Moderate activity (min ⋅ week^–1^)	125.0 ± 173.5	222.0 ± 216.6	0.196
Walking (min ⋅ week^–1^)	284.3 ± 280.4	207.7 ± 105.3	0.332
PA (METs h^–1^ ⋅ week^–1^)	32.7 ± 26.0	42.1 ± 18.4	0.277

*Data are expressed as Mean ± Standard Deviation. HR = heart rate; SBP = systolic blood pressure; DBP = diastolic blood pressure; PA = physical activity.*

Forced vital capacity (FVC) and FEV_1_ were lower in the PE group than in healthy controls. However, the FEV_1_/FVC ratios did not differ between the two groups (Table 2).

**TABLE 2:** Pulmonary function test findings of participants investigated.

	Pectus excavatum	Healthy controls	p=
FVC (% predicted)	90.4 ± 14.0	104.4 ± 17.0	0.021
FEV_1_ (% predicted)	96.7 ± 12.4	111.3 ± 15.7	0.009
FEV_1_/FVC (% predicted)	92.5 ± 3.9	93.9 ± 4.8	0.369

*Data are expressed as Mean ± Standard Deviation. FVC = forced vital capacity; FEV_1_ = forced expiratory volume in first second of expiration.*

Maximal heart rate was 184 ± 13 bpm in patients with pectus excavatum and 186 ± 7 bpm in controls (ns), indicating an equivalent maximal exercise effort in the 2 groups. Conversely, SBP at peak exercise showed lower values in the PE than in the HC group.

**FIGURE 1:**
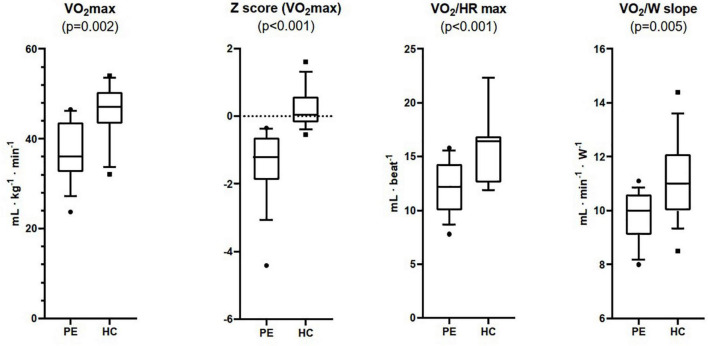
Maximum oxygen uptake (VO_2_ max), Z Score for VO_2_ max, maximum oxygen pulse (VO_2_/HR max), and work efficiency (VO_2_/W slope) measured at Cardiopulmonary Exercise Testing in patients with pectus excavatum (PE, *n* = 15), and healthy controls (HC, *n* = 15).

VO_2_ max was lower in the PE group than in HC both in absolute (37.2 ± 6.6 vs. 45.4 ± 6.4 mL⋅Kg^–1^⋅min^–1^, *p* < 0.05) and in percentage of the predicted value (76.9 ± 12.5% vs. 100.3 ± 8.3%, *p* < 0.05) (Figure 1). Average z score of VO_2_ max was -1.3 ± 1.0 (95% CI −1.97 to −0.58) and 0.2 ± 0.6 (95% CI −0.20 to 0.93) respectively. Similarly, O_2_ pulse at exercise peak (VO_2_/HR max) was lower in the PE group than in HC both in absolute (12.1 ± 2.4 vs. 16.2 ± 3.6 mL⋅beat^–1^, respectively, *p* < 0.05) and in percentage of the predicted value (87.5 ± 12.7% vs. 112.3 ± 10.6%, *p* < 0.05) (Table 3). Likewise work efficiency, expressed by the VO_2_/W slope, was lower in the PE than HC group (Table 3).

**TABLE 3:** Cardiopulmonary Exercise Testing Data.

	Pectus excavatum	Healthy controls	p=
Peak HR (bpm)	184.3 ± 13.3	185.8 ± 7.4	0.712
Peak HR (% of the predicted value)	90.1 ± 6.5	91.0 ± 3.4	0.650
SBP peak (mm Hg)	136.0 ± 13.0	148.7 ± 12.5	0.011
DBP peak (mm Hg)	59.7 ± 6.7	49.3 ± 8.8	0.001
Peak Power (W)	177.3 ± 45.2	220.1 ± 52.8	0.024
Peak Power (% of the predicted value)	75.5 ± 16.8	96.0 ± 12.2	0.001
VO_2_max (mL ⋅ kg^–1^ ⋅ min^–1^)	37.2 ± 6.6	45.4 ± 6.4	0.002
VO_2_max (% of the predicted value)	76.9 ± 12.5	100.3 ± 8.3	0.001
Z Score for VO_2_max	−1.3 ± 1.0	0.2 ± 0.6	0.001
VO_2_/HR max (mL ⋅ beat^–1^)	12.1 ± 2.4	16.2 ± 3.6	0.001
VO_2_/HR max (% of the predicted value)	87.5 ± 12.7	112.3 ± 10.6	0.001
VO_2_ max ⋅ SBP max (mL ⋅ kg^–1^ ⋅ min^–1^ ⋅ mm Hg)	5082.4 ± 1188.8	6797.9 ± 1307.4	0.001
VO_2_/W slope (mL ⋅ min^–1^ ⋅ W^–1^)	9.8 ± 0.9	11.1 ± 1.5	0.005

*Data are expressed as Mean ± Standard Deviation. HR = heart rate; SBP = systolic blood pressure; DBP = diastolic blood pressure; W = Watt; VO_2_ = volume of oxygen uptake.*

The relationships of cardiopulmonary performance (expressed by VO_2_ max), stroke volume at peak exercise (expressed as O_2_ pulse, VO_2_/HR max), and work efficiency (VO_2_/W slope) (all dependent variables) with average PA per week measured with IPAQ (independent variable) were then investigated at multivariable linear regression analysis adjusted for sex, BMI, FEV_1_%, and presence of pectus excavatum (Table 4).

**TABLE 4:** Predictors of VO_2_ max, and VO_2_/HR max (dependent variables) at multivariable linear regression analysis.

Independent variables	VO_2_max		VO_2_/HR max	
	**B (95% Cl)**	**p=**	**B (95% Cl)**	**p=**

Sex (men)	9.70 (6.06 to 13.35)	0.001	4.56 (2.68 to 6.44)	0.001
Body Mass Index (kg/m^2^)	−1.91 (−2.81 to −1.00)	0.001	−0.08 (−0.55 to 0.38)	0.716
FEV_1_%	0.19 (0.05 to 0.32)	0.009	0.06 (−0.01 to 0.13)	0.104
Diagnosis of PE (no)	9.24 (5.48 to 12.99)	0.001	2.73 (0.79 to 4.66)	0.008
PA (METs h^–1^ ⋅ week^–1^)	0.085 (0.010 to 0.160)	0.029	0.035 (−0.004 to 0.074)	0.073
R^2^	0.778		0.695	

*VO_2_ max = maximum oxygen uptake; VO_2_/HR max = maximum oxygen pulse; FEV_1_% = forced expiratory volume in first second of expiration,% predicted; PE = pectus excavatum; PA = physical activity.*

**FIGURE 2:**
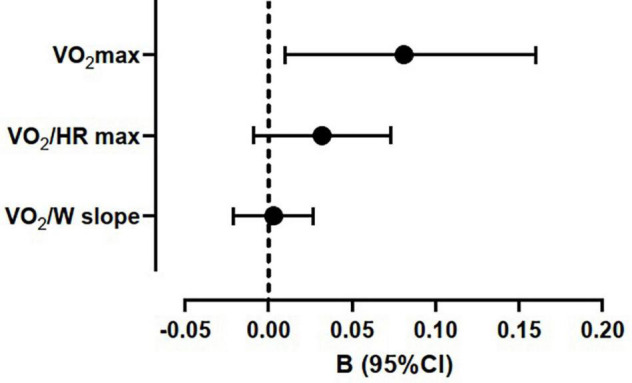
Regression coefficients of maximum oxygen uptake (VO_2_ max), maximum oxygen pulse (VO_2_/HR max), and work efficiency (VO_2_/W slope) with average weekly physical activity (METs h^–1^⋅week^–1^) at multiple linear regressions. The models were adjusted for sex, body mass index, forced expiratory volume in first second of expiration (% predicted), diagnosis of pectus excavatum, and physical activity (METs h^–1^⋅week ^–1^).

VO_2_ max recognized among its determinants METs h^–1^⋅week^–1^. Conversely, both VO_2_/HR max and VO_2_/W slope were not affected by METs h^–1^⋅week^–1^ at linear regression analysis, adjusted with the same model (Figure 2).

## Discussion

According to the present data, the average physical activity per week measured with the IPAQ questionnaire (Craig et al., 2003) was associated positively with a measurement of cardiopulmonary performance (VO_2_ max), whereas no association was observed with O_2_ pulse (VO_2_/HR max), a measure of stroke volume.

The relative role of psychological, anatomical and physiological alterations in determining functional impairment in PE is not easily discernible in the individual patient, although this point is crucial for the choice of the treatment strategy. A conservative strategy based on the promotion of PA may be useful to improve functional capacity, while the presence of an anatomical defect, with compression of the sternum resulting in a diastolic filling defect, could indicate a surgical choice. The results of this preliminary study seem to indicate that a cardiopulmonary test can help to assess the potential effectiveness of these two instruments. In patients with PE a reduced retrosternal space, leading to a diastolic dysfunction mainly in the right ventricle, could lead to a stroke volume reduction with a final impairment of cardiorespiratory fitness. In this study, O_2_ pulse, a measure of this mechanical parameter, was found to characterize the difference between PE and controls, independently of the estimation of PA or ventilatory function.

Cardiopulmonary performance expressed by VO_2_ max, also lower in PE compared to controls, instead also recognized among its determinants the average PA per week measured with the IPAQ. The promotion of PA in pectus excavatum, an important element in the treatment strategy of these patients, may improve cardiopulmonary performance, whereas stroke volume (O_2_ pulse) impairment that might be present in this condition seems to be independent from PA. A reduced O_2_ pulse at the peak of exercise could thus better address toward surgery.

Many studies have reported lower VO_2_ max in PE patients compared to controls (Jaroszewski et al., 2018; Obermeyer et al., 2018) and an increase in this parameter was observed after surgical repair (Maagaard and Heiberg, 2016; Obermeyer et al., 2018). Although physicians are very interested in cardiopulmonary test for this clinical condition, a consensus has not yet been established for the definition of a pathological threshold to address a patient to surgery. There have been few studies reporting reference values for submaximal and maximal cardiorespiratory fitness parameters, that may have interesting prognostic significance, such as O_2_ pulse, VO_2_/work slope, and the oxygen uptake efficiency slope. The main attention has been focused on VO_2_ max and O_2_ pulse, with limited attention to the great complexity of parameters offered by a cardiopulmonary test and their possible interaction with PA.

From our knowledge, this is the first attempt to correlate PA levels with cardiopulmonary test parameters in adolescents with PE.

Minimally invasive Nuss procedure for PE was consistently reported to improve stroke volume independently from the distance between surgery and functional evaluation due to the acute relief of constrained diastolic filling (Morshuis et al., 1994; Haller and Loughlin, 2000; Sigalet et al., 2007; Neviere et al., 2011, 2013; Kelly et al., 2013; Maagaard et al., 2013; O’Keefe et al., 2013). The increase in VO_2_ max was conversely not recorded when the assessment was carried out in the first months after surgery (Sigalet et al., 2007; O’Keefe et al., 2013). The present pilot study was dedicated to adding information in the decisional process between surgery and conservative strategies including the promotion of physical activity. Training program should be encouraged to increase cardiopulmonary performance expressed as VO_2_ max. However, a marked reduction in systolic stroke at peak exercise (expressed by the reduction in pulse O2) might instead suggest the surgical option.

For VO_2_ max the limit of normality was usually set at 80% of the predicted value (Blanchard et al., 2018). This lower limit seems to have been borrowed from the literature in forced spirometry (Miller et al., 2011), but its validity in categorizing normal cardiorespiratory fitness is lacking. This point, as well as the large inter-participant variability of VO_2_ max, has led to the possibility of using the Z-Score value, a statistic tool that indicates how far and in what direction (positive or negative) a measured value deviates from the population mean, expressed in units of the population SD. The limit of the ± 2 Z score directly identifies the 95% limit of a normal distribution and has often been chosen as the cut point to distinguish healthy from unhealthy. As a standardized measure, the Z-Score allows comparisons across different ages, genders and measurements. Most importantly, differently from adults, the use of the Z-Score allows more accurate assessments in children and adolescents, where small changes in age or pubertal stage produce big differences in sub-populations.

This is a pilot study with several limitations. First of all, an important limitation of the study is the limited number of participants studied. This limited number could be responsible for a type II error (beta) with the lack of correlation between PA and O_2_ pulse. The study must be considered a proof of principle study that may offer a perspective, although requiring confirmation. Second, there is a lack of accurate investigation of the diastolic function and of the morphological parameters used to characterize these patients, such as the echocardiographic indexes and the Haller index. Third, PA was measured with a questionnaire (IPAQ) and not an objective tool as accelerometer. Fourth, in the current study, differences in perceptions of body image and social appearance were not assessed. It may be important to assess the psychological impact by comparing the patient group with controls.

## Conclusion

In conclusion, average PA per week measured with the IPAQ was found to be associated with cardiopulmonary performance expressed as VO_2_ max, whereas no relationship was observed with stroke volume at peak exercise, expressed by O_2_ pulse.

## Data Availability Statement

The raw data supporting the conclusions of this article will be made available by the authors, without undue reservation.

## Ethics Statement

The studies involving human participants were reviewed and approved by Comitato Etico Regionale per la Sperimentazione Clinica della Regione Toscana. Written informed consent to participate in this study was provided by the participants’ legal guardian/next of kin.

## Author Contributions

PAM: conceptualization and writing—original draft preparation. LC and AP: methodology. AMo: formal analysis. LC, AP, AMe, MG, and FF: investigation. All authors have read and agreed to the published version of the manuscript.

## Conflict of Interest

The authors declare that the research was conducted in the absence of any commercial or financial relationships that could be construed as a potential conflict of interest.

## Publisher’s Note

All claims expressed in this article are solely those of the authors and do not necessarily represent those of their affiliated organizations, or those of the publisher, the editors and the reviewers. Any product that may be evaluated in this article, or claim that may be made by its manufacturer, is not guaranteed or endorsed by the publisher.
